# Design, Synthesis, Docking Studies and Monoamine Oxidase Inhibition of a Small Library of 1-acetyl- and 1-thiocarbamoyl-3,5-diphenyl-4,5-dihydro-(1*H*)-pyrazoles

**DOI:** 10.3390/molecules24030484

**Published:** 2019-01-29

**Authors:** Paolo Guglielmi, Simone Carradori, Giulio Poli, Daniela Secci, Roberto Cirilli, Giulia Rotondi, Paola Chimenti, Anél Petzer, Jacobus P. Petzer

**Affiliations:** 1 Dipartimento di Chimica e Tecnologie del Farmaco, Sapienza University of Rome, P.le A. Moro 5, 00185 Rome, Italy; paolo.guglielmi@uniroma1.it (P.G.); daniela.secci@uniroma1.it (D.S.); giulia.rotondi@uniroma1.it (G.R.); paola.chimenti@uniroma1.it (P.C.); 2 Department of Pharmacy, “G. D’Annunzio” University of Chieti-Pescara, Via dei Vestini 31, 66100 Chieti, Italy; 3 Department of Pharmacy, Università di Pisa, via Bonanno 6, 56126 Pisa, Italy; giulio.poli@unipi.it; 4 Centro nazionale per il controllo e la valutazione dei farmaci, Istituto Superiore di Sanità, Viale Regina Elena 299, 00161 Rome, Italy; roberto.cirilli@iss.it; 5 Pharmaceutical Chemistry, School of Pharmacy and Centre of Excellence for Pharmaceutical Sciences, North-West University, Potchefstroom 2520, South Africa; 12264954@nwu.ac.za (A.P.); jacques.petzer@nwu.ac.za (J.P.P.)

**Keywords:** pyrazoline, monoamine oxidase, enantioseparation, molecular modelling, prenyl

## Abstract

New *N*-acetyl/*N*-thiocarbamoylpyrazoline derivatives were designed and synthesized in high yields to assess their inhibitory activity and selectivity against human monoamine oxidase A and B. The most important chiral compounds were separated into their single enantiomers and tested. The impact of the substituents at N1, C3 and C5 positions as well the influence of the configuration of the C5 on the biological activity were analyzed. Bulky aromatic groups at C5 were not tolerated. *p*-Prenyloxyaryl moiety at C3 oriented the selectivity toward the B isoform. The results were also corroborated by molecular modelling studies providing new suggestions for the synthesis of privileged structures to serve as lead compounds for the treatment of mood disorders and neurodegenerative diseases.

## 1. Introduction

Monoamine oxidases, (MAOs) have been widely recognised as important pharmacological targets for the treatment of mood disorders (anxiety, depression) and neurodegeneration (Parkinson’s disease, PD) as the results of their effects on monoamine metabolism and level [1,2]. Two isoforms (MAO-A and MAO-B) have been characterized based on structural homology, tissue localization, substrate and inhibitor selectivity, active site differences and catalytic efficiency [3]. Both of them could be the target of selective inhibitors acting as reversible or irreversible agents. The interest in these enzymes brought an increased attention in the last years to a high number of licensed compounds [4,5,6,7,8]. Among several chemical scaffolds, pyrazoline derivatives have been extensively studied in the past as a valid scaffold for the design of human monoamine oxidase (hMAOs) inhibitors [1,2,3,9]. Our research group has explored the effect of changes involving mainly positions (N1, C3 and C5) of pyrazoline ring on MAO inhibition [10,11,12,13]. In particular, the N1 position was substituted with an (un)substituted phenyl ring, or acetyl, propanoyl and thiocarbamoyl moieties [14]. These attempts showed that the phenyl, 4-chlorophenyl and propanoyl groups negatively affect inhibitory activity of the hMAO enzymes compared with the acetyl or thiocarbamoyl one. The C3 and C5 positions have been substituted with aryl/heteroaryl moieties. However, pyrazolines substituted only at the N1 and C3 positions have also been tested [15]. The aryl system on the C3 and C5 positions has been substituted with different groups (halogens, hydroxy, methoxy, methyl) to evaluate how they affect MAO inhibitory activity [14]. Furthermore, the presence of the C5 chiral centre results in the presence of two enantiomers, which could bind and interact differently with the two enzymes.

**Figure 1:**
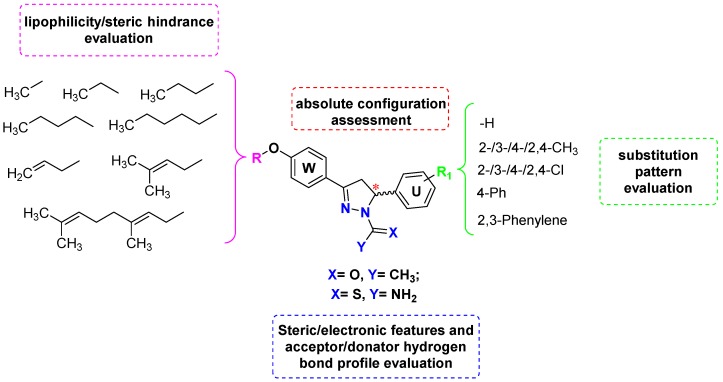
Design of the new pyrazoline-based human monoamine oxidase (hMAO) inhibitors.

With the purpose to expand our knowledge on the structure-activity relationships (SARs) for hMAOs inhibition by pyrazolines, we have designed and tested 18 new or previously described compounds (**P1**–**P18**) based on the 1,3,5-substituted pyrazoline scaffold (Figure 1). The N1 position of pyrazoline ring was substituted with either the thiocarbamoyl (derivatives **P1**–**P6**) or the acetyl (derivatives **P7**–**P18**) moiety. These groups possess different steric/electronic properties, and differ in their hydrogen bonds acceptor/donor potential. Position C3 of pyrazoline ring was functionalized with a phenyl ring (shown as **W** Figure 1), which was, in turn, substituted on the *para*-position with saturated/unsaturated alkyloxy groups. We attempted to understand if the increasing lipophilicity associated with the alkyl chain length affected the inhibitory activity. Pursuing this idea, prenyl and geranyl moieties, containing respectively one and two isoprenoid units, have also been inserted. The insertion of an isoprenoid unit is a post-translational modification that is, among the others, able to affect the interaction of proteins with membrane cells [16,17]. Therefore, the presence of prenyl and geranyl groups could enhance the ability of these inhibitors to interact with the outer mitochondrial membrane where the hMAO-A and hMAO-B are located. The position C5 of pyrazoline was substituted with a phenyl ring (shown as **U** in Figure 1) containing substituents with diverse steric/electronic features as well as the unsubstituted bulky naphthyl group. Finally, HPLC chiral resolution of some derivatives (**P1**–**P5**) was carried out to evaluate how the chiral properties of the single enantiomers influence the inhibitory activity against hMAO enzymes [18].

## 2. Results and Discussion

### 2.1. Chemistry and HPLC Enantioseparation

**Scheme 1:**
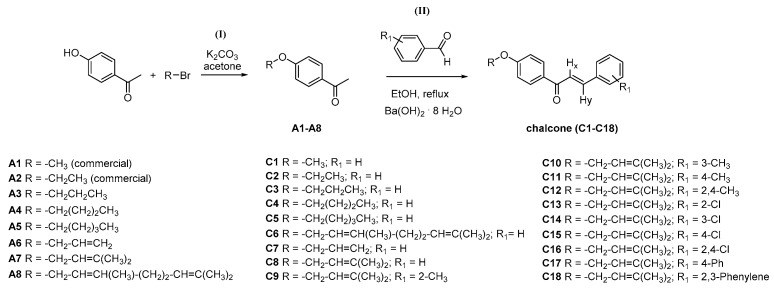
Synthesis and structures of acetophenones **A1**–**A8** and chalcones **C1**–**C18**.

**Scheme 2:**
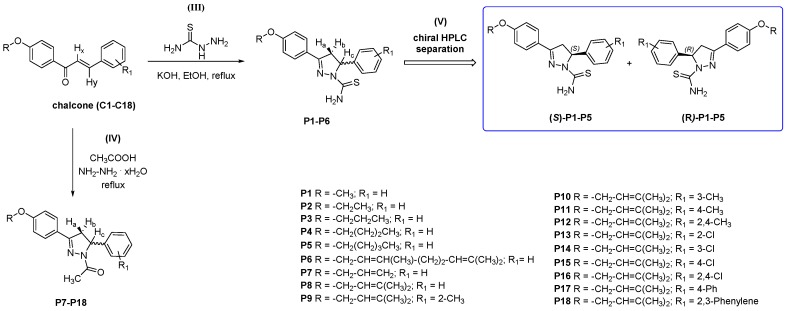
Synthesis and structures of compounds **P1**–**P18**.

The pyrazoline derivatives **P1**–**P18** were synthesized according to the protocol outlined in Scheme 1 and Scheme 2, using a synthetic approach via chalcone. This strategy has been extensively used in the past by our and other research groups, in order to obtain 1,3,5-substituted pyrazolines [11,19,20,21,22]. The first reaction of this multi-step procedure was the synthesis of *para*-substituted acetophenones (Scheme 1, **I**). In particular, 4-hydroxyacetophenone was reacted with different brominated compounds in order to obtain derivatives containing: (i) linear alkyl chain with increasing dimensions (**A1**–**A5**), (ii) unsaturated alkyl chain (**A6**) and (iii) unsaturated/branched alkyl chain (**A7**–**A8**). Acetophenones **A1**–**A2** were commercially available, while the syntheses of **A3**–**A8** were performed in acetone at room temperature, in the presence of dried potassium carbonate [23]. The so obtained acetophenones were used for the next Claisen–Smith condensation reaction (Scheme 1, **II**). This synthesis was performed in ethanol at reflux, using barium hydroxide (Ba(OH)_2_ × 8H_2_O) as heterogeneous catalyst. In these conditions, the ketone is adsorbed on basic sites generating the carbanion which will react with the aldehyde [24]. In solid–liquid reaction environments, the number of accessible sites of the solid, which are able to catalyze the reaction, influences the catalytic efficiency. Increasing the superficial area of the solid can increase the number of these “active sites” located on the external, and so accessible, part of the Ba(OH)_2_ particles. Since the superficial area is influenced by particle dimensions, we ground barium hydroxide prior to the synthesis. By means of this reaction, we attained the chalcones **C1**–**C18** in moderate to high yields. NMR and IR spectroscopic data confirmed the intermediate structure. In particular, NMR spectra exhibited the presence of the two alkene protons (shown as H_x_ and H_y_ in Scheme 1) contained in the 1,3-diaryl-2-propen-1-one system (chalcone). Furthermore, the coupling constants between these two atoms were around 15–16 Hz, hallmark of the interaction between protons belonging to alkene system in the (*E*) configuration. The IR spectra (neat) for derivatives **C1**–**C18** showed stretching absorption bands at approximately 2910 cm^−1^ due to the stretching of C_sp3_-H, at 1600 cm^−1^ due to the stretching of C=O and at 1439 cm^−1^ for the C=C. At 1171 cm^−1^ we observed a signal related to the C-O stretching, while other bands at 829, 820, and 775 cm^−1^ were attributed to C_sp2_-H bending.

Then, two series of pyrazoline derivatives were obtained through different routes already reported. *N*1-thiocarbamoylated derivatives (**P1**–**P6**) were synthesized through the reaction between the proper chalcone with an excess of thiosemicarbazide and potassium hydroxide, in ethanol at reflux (Scheme 2, **III**) [20]. Instead, the *N*1-acetylated derivatives (**P7**–**P18**) were obtained by means of the reaction between the chalcone with excess of hydrazine in acetic acid, at reflux conditions (Scheme 2, **IV**) [10]. The formation of the pyrazoline ring were confirmed by spectroscopic outcomes, exhibiting the typical NMR profile of 3,5-disubstituted pyrazolines. In fact, the two protons bound on C(4) of pyrazoline ring (H_a_ and H_b_ in Scheme 1) are diastereotopic and at 400 MHz resonated as a pair of doublets of doublets at ~3.00 ppm and ~3.74 ppm. The C(5) proton (H_c_ in Scheme 1) was observed at ~5.71 ppm as a doublets of doublets owing to coupling with the two vicinal protons of the methylene group which are non-magnetically equivalent [25]. Further evidence about the final compounds structure, was also obtained by means of IR spectroscopy. In general, the IR spectrum (neat) for derivatives **P1**–**P6** showed stretching absorption bands at approximately 3486 and 3354 cm^−1^ for NH, at 2979 cm^−1^ due to the stretching of C_sp3_-H, at 1611 cm^−1^ for the C=N, at 1571 cm^−1^ for the C=S stretching of the *N*-thiocarbamoylated derivatives, and 1495 cm^−1^ for C=C. At 1252 cm^−1^ we observed a signal related to the C-N stretching, while we assigned the two bands at 1170 and 1050 cm^−1^ to the C-O stretching. Other bands at 825, 760, 695 cm^−1^ were attributed to C_sp2_-H bending. The IR spectrum (neat) for derivatives **P7**–**P18** showed little differences compared to **P1**–**P6** compounds, as the disappearance of the stretching absorption bands of NH and the presence of the carbonyl moiety signal, instead of thiocarbamoyl one. These compounds showed absorption bands at 2945 cm^−1^ due to the stretching of C_sp3_-H and at 1770 and 1622 cm^−1^ for the stretching of C=O. Additional bands were observed at 1595 cm^−1^ for the C=N, and at 1475 cm^−1^ for C=C. The C-N stretching exhibited adsorption bands at 1247 cm^−1^. Other bands at 817, 741, 699 cm^−1^ were attributed to C_sp2_-H bending.

The presence of a stereogenic centre at C5 of the pyrazoline ring led us to evaluate the potential stereospecificity in the MAO inhibitory activity. As indicated in Scheme 2 (**V**), the compounds **P1**–**P5** were selected and mg-quantities of their pure enantiomeric forms were easily isolated by semipreparative enantioselective HPLC on the Chiralpak AD column using pure ethanol elution mode [26]. As previously described [26,27] the first eluting enantiomer of **P1**–**P5** has an (*S*)-configuration and the second eluting enantiomer an (*R*)-configuration.

### 2.2. hMAO-A and hMAO-B Inhibition Studies

The synthesized compounds **P1**–**P18** were evaluated as hMAO inhibitors using the recombinant hMAOs as enzyme sources and kynuramine as substrate. A fluorometric method based on the Amplex^®^ Red reagent (λ_ex_ = 560 nm; λ_em_ = 590 nm) was used to measure hMAO activity. In this horseradish peroxidase-linked assay, Amplex^®^ Red reacts with hydrogen peroxide that is produced by the MAO catalytic cycle to yield a fluorescent product, resorufin [28]. By thus recording the MAO-catalyzed formation of hydrogen peroxide in the presence of different concentrations of a test inhibitor, sigmoidal plots of enzyme activity versus the logarithm of inhibitor concentration were constructed from which IC_50_ values were estimated (GraphPad Prism 5). All IC_50_ values were measured in triplicate and are expressed as the mean ± standard deviation (SD). The inhibitory activities of compounds **P1**–**P18** are summarized in Table 1 along with selectivity index (SI) values which are the ratio of IC_50_(hMAO-A)/IC_50_(hMAO-B). SI < 1 indicates selectivity for MAO-A, whereas SI > 1 indicates selectivity for the MAO-B isoform.

As previously stated, the compounds **P1**–**P5** were resolved by chiral HPLC [26] and tested as single enantiomers. In this way, we attempted to examine the chiral recognition of these compounds by hMAO-A and hMAO-B. The **W**-phenyl ring (see Figure 1) of compounds **P1**–**P5** was *para-*substituted with linear alkyloxy chains of increasing length (from one to five carbon atoms), while position N1 was substituted with a thiocarbamoyl group.

The **P1** enantiomers substituted with the methoxyl were selective for hMAO-A, but exhibited inhibitory activity in the high micromolar range (hMAO-A: IC_50_ (*S*)-**P1** = 46.6 µM; IC_50_ (*R*)-**P1** = 50.6 µM), without activity against hMAO-B at the maximal tested concentration of 100 µM. The IC_50_ values of the two enantiomers were almost equal, showing that the hMAO-A enzyme likely interacted with them in a similar manner. The ethoxy-substituted derivatives gave comparable results with the **P2** enantiomers being devoid of inhibition activity against hMAO-B. Similar to the **P1** enantiomers, the **P2** enantiomers exhibited weak inhibition of hMAO-A (hMAO-A: IC_50_ (*S*)-**P2** = 32.3 µM; IC_50_ (*R*)-**P2** = 33.9 µM) with virtually the same IC_50_ values. Differences arose when the length of the bound alkyl group was increased. The **P3** enantiomers containing the propyloxyl group exhibited improved inhibition activity against hMAO-A compared to the **P1** or **P2** derivatives (hMAO-A: IC_50_ (*S*)-**P3** = 9.59 µM; IC_50_ (*R*)-**P3** = 8.00 µM). As for the **P1** or **P2** derivatives, hMAO-A did not show preference for one of the two enantiomers. However, the presence of inhibitory activity against hMAO-B for (*S/R*)-**P3** was observed, with IC_50_ values depending on the absolute configuration. In fact, the (*S*)-enantiomer was less potent than the (*R*)-enantiomer (hMAO-B: IC_50_ (*S*)-**P3** = 42.5 µM; IC_50_ (*R*)-**P3** = 2.77 µM), which resulted in SI values for (*S*)-**P3** and (*R*)-**P3** of 0.23 and 2.89, respectively. Considering this finding, it may be concluded that hMAO-B is able to recognize (*R*)-**P3** as the eutomer.

Further increase of the alkyl chain length led to the compounds **P4** and **P5**, containing respectively butoxyl and pentoxyl moieties (Table 1). The **P4** enantiomers displayed inhibitory activity towards both hMAO isoforms. For hMAO-A, the IC_50_ of the (*R*)-enantiomer was 10-fold higher than that observed for the (*S*)-enantiomer (hMAO-A IC_50_: (*S*)-**P4** = 39.2 µM; IC_50_ (*R*)-**P4** = 3.63 µM). hMAO-B inhibition followed a similar trend with the inhibitory activity of (*R*)-**P4** being in the submicromolar range (hMAO-B: IC_50_ (*R*)-**P4** = 0.38 µM), while (*S*)-**P4** was significantly less potent (hMAO-B: IC_50_ (*S*)-**P4** = 5.03 µM). Therefore, the (*R*)-**P4** enantiomer was the eutomer, showing the highest potency inhibition against both hMAO-A and hMAO-B. Comparable results were obtained for the **P5** enantiomers, bearing the pentoxyl group. In this instance, the (*R*)-enantiomer showed inhibitory activities towards hMAO-A and hMAO-B that are 18- and 6-fold higher than those of the (*S*)-enantiomer [(*R*)-**P5** IC_50_ hMAO-A = 3.03 µM; IC_50_ hMAO-B = 0.44 µM]. These outcomes showed that when increasing the length of the alkyl chain, both enzymes are able to recognize one of the two optical isomers as the eutomer and the preferred absolute configuration appears to be (*R*). Finally, the substitution with the geranyl moiety was considered in order to determine how a branched chain, containing unsaturation and longer length compared to the other derivatives tested, affected the inhibition activity and/or the selectivity. The results showed that compound **P6** was ineffective as an inhibitor of hMAO-A, retaining activity in the high micromolar range only against hMAO-B (hMAO-B: IC_50_ **P6** = 54.1 µM). Therefore, the excessive increase of the alkyl chain dimensions was detrimental for inhibition potency, although selectivity for hMAO-B was obtained.

Derivatives **P7**–**P18**, which are substituted on the N1 position with the acetyl group, have also been tested. Except for compound **P7**, bearing an allyloxy functional group, all derivatives possessed *para*-prenyloxy substitution on the phenyl ring bound at the position 3 of pyrazoline ring (**W** ring in Figure 1). However, substituents on the phenyl bound at the position 5 of pyrazoline (**U** ring in Figure 1) were varied in order to obtain preliminary SARs. Compound **P7** inhibited both isoforms with a slight preference for hMAO-B and IC_50_ values in the low micromolar range were recorded (**P7**, IC_50_ hMAO-A = 39.3 µM; IC_50_ hMAO-B = 14.5 µM; SI = 2.72). Compound **P8** was the simplest compound endowed with prenyl group, bearing an unsubstituted phenyl ring on position 5 of pyrazoline ring (the **U** phenyl). This compound exhibited activity against hMAO-B in the low micromolar range, without activity for hMAO-A (**P8**, IC_50_ hMAO A > 100 µM; IC_50_ hMAO-B = 2.29 µM; SI = 43.67).

The addition of substituents on the **U** phenyl ring affected both activity and selectivity. Compounds **P9**–**P11** were substituted with methyl groups at respectively the *ortho*, *meta* and *para* positions, while compound **P12** contained the 2,4-dimethyl substituted phenyl ring. Derivative **P9** showed preference for hMAO-A with an inhibitory activity that was 3-fold higher than that observed for hMAO-B (**P9**, IC_50_ hMAO-A = 9.13 µM; IC_50_ hMAO-B = 27.3 µM; SI = 0.33). Shifting of the methyl group from *ortho* to *meta* position (**P10**) resulted in a change of selectivity along with an enhancement of inhibitory activity towards hMAO-B, with an IC_50_ value in the low micromolar range (**P10**, IC_50_ hMAO-A = 86.8 µM; IC_50_ hMAO-B = 3.22 µM; SI = 26.96). For compound **P11**, bearing the methyl group in the *para* position, hMAO-A and hMAO-B inhibition was reduced compared to **P10** (**P11**, IC_50_ hMAO-B = 12.2 µM). The 2,4-dimethyl substituted derivative **P12** exhibited weak inhibitory activity against both isoforms, with only slight inhibition recorded for hMAO-A (**P12**, IC_50_ hMAO-A = 80.6 µM). The presence of 2,4-disubstituted phenyl system at the C5 position thus is detrimental for the hMAO inhibition. Similarly, **P16**, bearing the 2,4-dichloro substituted phenyl ring, did not inhibit either hMAO-A or hMAO-B. It may be concluded that a sterically hindered system on the C5 position of the pyrazoline ring negatively affects the inhibitory activity. This was demonstrated by **P17** and **P18**, which were substituted with bulky naphthyl and biphenyl groups, respectively, and are devoid of inhibition activity against both hMAO-A and hMAO-B.

Finally, compounds **P13**–**P15**, which were substituted with chlorine on the phenyl ring, have been evaluated. Derivative **P13**, the *ortho*-chloro substituted derivative, showed similar inhibitory activity against both isoforms (**P13**, IC_50_ hMAO-A = 5.74 µM; IC_50_ hMAO-B = 3.11 µM; SI = 1.85). **P15**, containing a *para*-chlorophenyl, showed a similar profile, with an inhibitory activity against both hMAO A and hMAO-B, although less potent than **P13** (**P15**, IC_50_ hMAO-A = 12.1 µM; IC_50_ hMAO-B = 11.0 µM; SI = 1.11). Among the chloro-substituted molecules, the best inhibitory activity against hMAO B was observed when the substituent was placed in the *meta* position, as previously seen for methyl substituted compounds (**P9**–**P12**). In fact, **P14** displayed inhibition activity against hMAO-B in the low micromolar range, but it also retained a good activity on hMAO-A (**P14**, IC_50_ hMAO-A = 4.13 µM; IC_50_ hMAO-B = 1.08 µM; SI = 3.82).

In the light of the above, some considerations regarding the properties (dimensions and lipophilicity) of the alkyl chains bound to the **W** ring can be done. We already stated that the excessive size of this substituent was detrimental for the inhibitory activity, as observed for compound **P6**. However, the increase of the alkyl chain length (compounds **P1**–**P5**) also involves the enhancement of lipophilicity leading, at least for the (*R*)-enantiomers, in the improvement of the affinity against both the isoforms (see Table 1). From these data it would seem that the gain in lipophilicity could mitigate the increase of the size, until the group is too big for the active site; in this instance, the steric hindrance prevails on hydrophobic interactions impairing the inhibitory activity. The branched prenyl moiety (**P8**–**P18**) could be the perfect compromise with a good balance between lipophilicity and steric hindrance, improving the selectivity against MAO-B, without impairing the inhibitory activity. This is consistent with the bigger hydrophobic pocket observed in the binding site of MAO B, where the lipophilic chains of the ligands could be better accommodated than into MAO-A, as suggested by docking studies. Considering the shared scaffold within compounds **P1**–**P5** and the dimensions of the prenyl group, we can assume that also for derivatives **P8**–**P18**, MAO-B isozyme could exhibit chiral recognition for (*R*)-enantiomers. The use of racemic mixture for the biological evaluation could reduce the real inhibitory activity due to the competition between the two enantiomers for the same active site. Therefore, the results obtained for prenylated derivatives are worthwhile of further investigation.

### 2.3. Docking Studies

**Figure 2:**
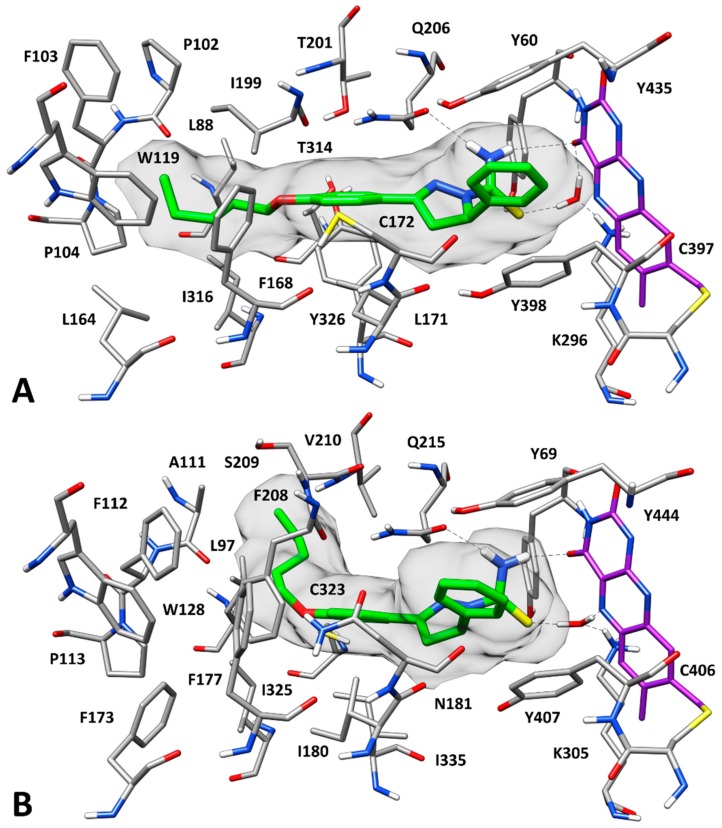
Predicted binding mode of (*R*)-**P5** into hMAO-B (**A**) and hMAO-A (**B**). For clarity, only the flavin group of the cofactor is shown in purple. The molecular surface of the ligand is shown in gray.

With the aim of providing a rational interpretation of the results obtained from the biological evaluation of the pyrazoline derivatives, molecular modeling studies including robust docking analyses and energy minimizations in explicit water environment were performed (see Materials and Methods for details). Initially, compound (*R*)-**P5**, which showed submicromolar activity against hMAO-B and low micromolar potency against hMAO-A, was subjected to the computational protocol in order to evaluate its binding mode into the catalytic site of the two enzymes. As shown in Figure 2A, the ligand well fits the long hydrophobic cavity constituting the substrate binding site of hMAO-B and forms several different lipophilic interactions with the protein residues. In particular, the terminal phenyl ring linked to the pyrazoline core is sandwiched between Y398 and Y435, forming π–π stacking interactions with these two residues and a T-shaped stacking with the flavin portion of FAD cofactor. The central phenyl ring of the inhibitor forms hydrophobic interactions with L171, C172, I199 and Y362, while the pentoxyl chain takes lipophilic contacts with several residues such as F103, P104, W119 and I316, which form a long hydrophobic pocket placed at the end of the binding cavity. Moreover, the thioamide moiety of the ligand anchors the compound to the enzyme binding site through a network of H-bonds. Precisely, two direct H-bonds are formed with the side chain of Q206 and the flavin portion of the cofactor, while a water-bridged interaction is observed among the ligand, the cofactor and K296. The reduced potency of the ligand against hMAO-A compared to hMAO-B may be ascribed to weaker hydrophobic interactions. In fact, by analyzing the binding mode predicted for the inhibitor into hMAO-A (Figure 2B), it is possible to notice that, although the H-bond network with the cofactor, the conserved Q215 and the structural water molecule can still be established by the thioamide group of the ligand, the overall disposition of the compound into the binding pocket of the enzyme is different. This is due to the presence of few non-conserved residues that significantly change the shape of the binding cavity of the two enzyme isoforms. In fact, the presence of F173 and F208 in hMAO-A in place of L164 and I199 of hMAO-B, respectively, determines the closure of the hydrophobic pocket placed at the end of the binding cavity. In contrast, the absence of Y326 of hMAO-B, which is replaced by the less bulky I335 in hMAO-A, provides access to a short channel communicating with the solvent where the ligand disposes its pentoxyl chain. For this reason, the whole chain shows reduced lipophilic contacts with the protein residues and its terminal portions are slightly exposed to the solvent. For this reason, the whole chain shows reduced lipophilic contacts with the protein residues and its terminal portions are slightly exposed to the solvent. Moreover, the phenyl ring of the ligand linked to the pyrazoline core moves away from the cofactor, Y444 and Y407, thus forming weaker stacking interactions with the two residues and losing contact with FAD.

**Figure 3:**
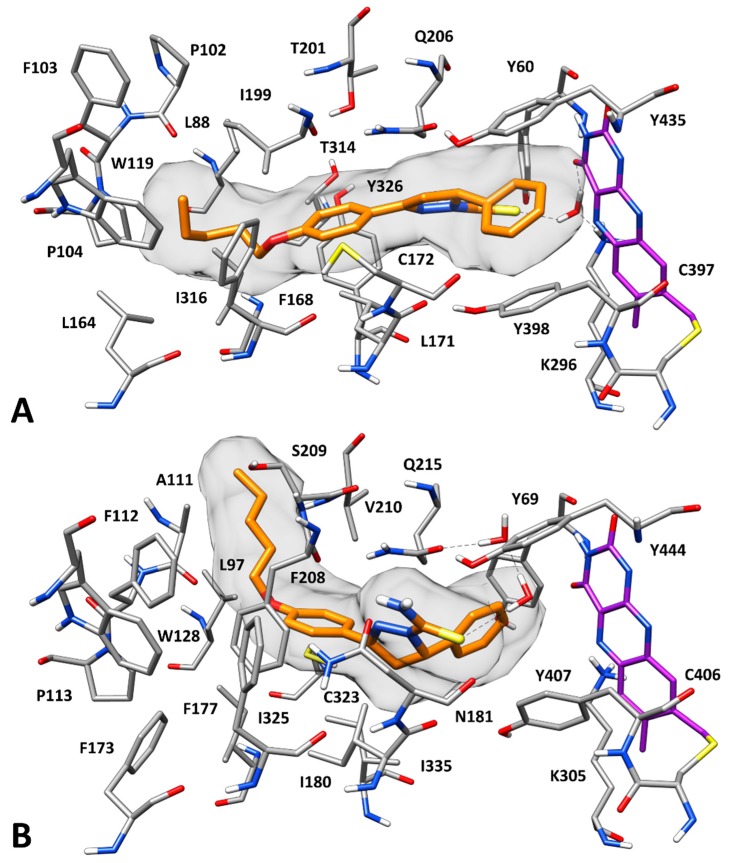
Predicted binding mode of (*S*)-**P5** into hMAO-B (**A**) and hMAO-A (**B**). For clarity, only the flavin group of the cofactor is shown in purple. The molecular surface of the ligand is shown in gray.

The same computational protocol was then applied to the (*S*)-enantiomer of the inhibitor, which showed a lower activity against both enzyme isoforms. The binding mode predicted for the compound into hMAO-B suggested that, due to the different stereochemistry of the pyrazoline ring, the ligand is not able to form the H-bond network with FAD and Q206 and only the water-bridged interaction with the carbonyl oxygen of the cofactor flavin group and the amino group of K296 is maintained (Figure 3A). However, the terminal phenyl ring of the inhibitor assumes a perfect orientation for the formation of strong π–π stacking interactions with Y398, Y435 and also the flavin group of FAD; moreover, the pyrazoline ring of the ligand can establish additional stacking interactions with the amide group of Q206. These interactions can probably compensate for the loss of the two H-bonds and thus limit the drop of activity of the ligand against hMAO-B with respect to its enantiomer. Differently, the inhibitory potency of (*S*)-**P5** on hMAO-A showed a more significant reduction, compared to (*R*)-**P5**. Accordingly, the ligand was predicted to assume a binding disposition characterized by an inverted orientation of the pyrazoline core, with respect to the binding mode of its (*R*)-enantiomer. For this reason, none of the H-bonds predicted for the (*R*)-enantiomer can be formed by compound (*S*)-**P5** and the thioamide group of the ligand can only form an H-bond with Q215 mediated by two water molecules. Moreover, the terminal phenyl ring of the ligand shows only a T-shaped stacking with Y69 in place of the strong π–π stacking with Y398, Y435 and FAD flavin group observed in the predicted binding mode into hMAO-B. Finally, since the position of the ligand is slightly shifted toward the end of the binding cavity, its pentoxyl chain protrudes more deeply into the short channel connected to the solvent and may thus form unfavorable interactions with water.

Based on the binding modes predicted for the two compounds (*S*)-**P5** and (*R*)-**P5** into hMAO-A and hMAO-B, few considerations can be derived. The thioamide moiety of the ligands is important for both the H-bond donor NH_2_ group and the H-bond acceptor sulfur atom, with this last portion that seems to be a fundamental feature. This is consistent with the observation that derivatives with the thioamide moiety replaced by an acetyl group maintain micromolar activity. The different disposition assumed by the alkoxyl chain of the ligands into hMAO-A and hMAO-B could explain why compounds with longer chains are more selective over hMAO-B than those bearing shorter chains like (*R*)-**P3**: a shorter chain would limit not only the favorable hydrophobic interactions with the lipophilic pocket of hMAO-B, but also the possible unfavorable interactions with the solvent when bound to hMAO-A, thus balancing the activity of the inhibitor toward the two enzyme isoforms. Finally, the inactivity of derivatives bearing bulky groups connected to the pyrazoline core such as **P16**–**18** is consistent with the limited space available in proximity of the flavin portion of FAD cofactor.

## 3. Materials and Methods

### 3.1. General

Solvents and reagents were used as supplied without further purification (Sigma-Aldrich^®^, Milan Italy). Where mixtures of solvents are specified, the stated ratios are volume:volume. Stuart^®^ melting point apparatus SMP1 (Cole-Parmer, Stone, United Kingdom,) has been used to measure the melting points (uncorrected). IR spectra were measured with a PerkinElmer Spectrum 100 FT-IR spectrophotometer (PerkinElmer, Waltham, MA, USA), equipped with universal total reflectance (ATR) accessory with absorption frequencies expressed in reciprocal centimeters. ^1^H- and ^13^C-NMR spectra were recorded at 400 and 101 MHz, respectively, on a Bruker spectrometer (Bruker, Billerica, MA, USA) using CDCl_3_ and DMSO-*d*_6_ as the solvents at room temperature. Chemical shifts are expressed as δ units (parts per millions) relative to the solvent signal. ^1^H spectra are reported as follows: *δ*_H_ (spectrometer frequency, solvent): chemical shift/ppm (multiplicity, J-coupling constant(s), number of protons, assignment). ^13^C spectra are reported as follows: *δ*_C_ (spectrometer frequency, solvent): chemical shift/ppm (assignment). Multiplets are abbreviated as follows: br—broad; s—singlet; d—doublet; t—triplet; q—quartet; m—multiplet. Coupling constants *J* are valued in Hertz (Hz). Purification on column chromatography was carried out using silica gel (high purity grade, pore size 60 Å, 230–400 mesh particle size). All the operations were monitored by TLC performed on 0.2 mm thick silica gel-aluminum backed plates (60 F254, Merck, Darmstadt, Germany). Visualization was carried out under ultra-violet irradiation (254 nm). Where given, systematic compound names are those generated by ChemBioDraw Ultra 12.0 following IUPAC conventions (PerkinElmer, Waltham, MA, USA). A Perkin-Elmer 240 B microanalyzer (PerkinElmer, Waltham, MA, USA) was used to determine elemental analyses for C, H, and N; analytical results were within ±0.4% of the theoretical values for all the tested compounds. Microsomes from insect cells containing recombinant hMAO-A and hMAO-B (5 mg protein/mL) and kynuramine dihydrobromide were obtained from Sigma-Aldrich. Amplex^®^ Red (10-acetyl-3,7-dihydroxyphenoxazine), horseradish peroxidase, (*R*)-deprenyl hydrochloride, pargyline hydrochloride and H_2_O_2_ (3%) were also from Sigma-Aldrich. For fluorescence spectrophotometry, a SpectraMax iD3 multi-mode microplate reader (Molecular Devices, San Jose, CA, USA) was employed.

### 3.2. General Synthesis for Acetophenone Derivatives ***A3**–**A8***

To a stirring solution of 1-(4-hydroxyphenyl)ethan-1-one (1 eq.) in anhydrous acetone (20 mL) freshly ground potassium carbonate (1.2 eq.) was added. 1-Bromoalkane (1.1 eq.) was added and the reaction stirred at room temperature for 24–48 h, under nitrogen atmosphere. The synthesis was quenched with 50 mL of ice/water and the resulting suspension was extracted with chloroform (3 × 20 mL). The organics were reunited, dried over sodium sulfate and concentrated in vacuo, to give the crude compounds. Purification by column chromatography on silica gel (ethyl acetate:*n*-hexane 1:4) gave the title compounds. Characterization data obtained for compounds **A3**–**A5** and **A7** were in accordance with [26], while for compound **A6** were in accordance with [23].

*1-(4-((3,7-dimethylocta-2,6-dien-1-yl)oxy)phenyl)ethan-1-one* (**A8**), yellow-orange oil (72% yield). ^1^H-NMR (400 MHz, CDCl_3_): δ 1.59 (s, 3H, CH_3_), 1.66 (s, 3H, CH_3_), 1.73 (s, 3H, CH_3_), 2.07–2.11 (m, 4H, 2 × CH_2_), 2.52 (s, 3H, CH_3_), 4.58 (d, *J* = 6.4 Hz, 2H, CH_2_), 5.05–5.08 (m, 1H, =CH), 5.44–5.47 (m, 1H, =CH), 6.91 (d, *J* = 8.8 Hz, 2H, Ar), 7.90 (d, *J* = 8.8 Hz, 2H, Ar).

#### 3.2.1. General Synthesis and Characterization Data for Chalcones **C1**–**C18**

To a stirring solution of the proper chalcone (1 eq.) in ethanol (10 mL) was added a dispersion of barium hydroxide (1 eq.) in ethanol (40 mL). The appropriate (un)substituted benzaldehyde (1 eq.), dissolved in ethanol (20 mL), was added dropwise and the reaction stirred at reflux for 24–72 h. The synthesis was quenched with 150 mL of ice/water and the resulting suspension filtered. The crude solid was washed with petroleum ether (2 × 20 mL) and *n*-hexane (2 × 20 mL) giving the title compounds without further purification requirements. For compound **C18** the suspension obtained after quenching with 150 mL of ice/water, was extracted with chloroform (3 × 20 mL). The organics reunited were dried over sodium sulphate and evaporated in vacuo. Purification by column chromatography on silica gel (ethyl acetate:*n*-hexane 1:4) gave the title compounds. Characterization data obtained for compounds **C1**–**C5**, **C7**–**C8** were in accordance with [26,30], while for compound **C15** were in accordance with [23].

*(E)-1-(4-((-3,7-dimethylocta-2,6-dien-1-yl)oxy)phenyl)-3-phenylprop-2-en-1-one* (**C6**), white solid (82% yield); mp 66–68 °C. ^1^H-NMR (400 MHz, DMSO-*d*_6_): δ 1.56 (s, 3H, CH_3_), 1.62 (s, 3H, CH_3_), 1.73 (s, 3H, CH_3_), 2.06 (br, 4H, 2 × CH_2_), 4.68 (d, *J* = 6 Hz, 2H, CH_2_), 5.06 (br, 1H, =CH), 5.44 (br, 1H, =CH), 7.08 (d, *J* = 8 Hz, 2H, Ar), 7.45 (br, 3H, Ar), 7.71 (d, *J*_xy_ = 16 Hz, 1H, H_x_), 7.88 (d, *J* = 3.6 Hz, 2H, Ar), 7.95 (d, *J*_xy_ = 15.6 Hz, 1H, H_y_), 8.16 (d, *J* = 8.4 Hz, 2H, Ar).

*(E)-1-(4-((3-methylbut-2-en-1-yl)oxy)phenyl)-3-(o-tolyl)prop-2-en-1-one* (**C9**), yellow solid (92% yield); mp 72–75 °C. ^1^H-NMR (400 MHz, CDCl_3_): δ 1.79 (s, 3H, CH_3_), 1.83 (s, 3H, CH_3_), 2.50 (s, 3H, CH_3_), 4.62 (d, *J* = 6.4 Hz, 2H, CH_2_), 5.52 (br, 1H, =CH), 7.01 (d, 2H, *J* = 8.8 Hz, Ar), 7.24-7.33 (m, 3H, Ar), 7.49 (d, *J*_xy_ = 15.6 Hz, 1H, H_x_), 7.71 (d, *J* = 7.6 Hz, 1H, Ar), 8.06 (d, *J* = 8.4 Hz, 2H, Ar), 8.12 (d, *J*_xy_ = 15.6 Hz, 1H, H_y_).

*(E)-1-(4-((3-methylbut-2-en-1-yl)oxy)phenyl)-3-(m-tolyl)prop-2-en-1-one* (**C10**), yellow solid (77% yield); mp 99–101 °C. ^1^H-NMR (400 MHz, DMSO-*d*_6_): δ 1.74 (s, 3H, CH_3_), 1.76 (s, 3H, CH_3_), 2.36 (s, 3H, CH_3_), 4.65–4.66 (m, 2H, CH_2_), 5.45 (br, 1H, =CH), 7.08 (d, *J* = 7.6 Hz, 2H, Ar), 7.26–7.27 (m, 1H, Ar), 7.33–7.35 (m, 1H, Ar), 7.65–7.72 (m, 3H, 2 × Ar + H_x_), 7.92 (d, *J*_xy_ = 16 Hz, 1H, H_b_), 8.16 (d, *J* = 7.6 Hz, 2H, Ar).

*1-(4-((3-methylbut-2-en-1-yl)oxy)phenyl)-3-(p-tolyl)prop-2-en-1-one* (**C11**): yellow solid (87% yield); 95–98 °C. ^1^H-NMR (400 MHz, DMSO-*d*_6_): δ 1.74 (m, 6H, 2 × CH_3_), 2.35 (s, 3H, CH_3_), 4.64 (s, 2H, CH_2_), 5.44 (s, 1H, =CH), 7.08–8.15 (m, 10H, 8 × Ar + 2 × =CH).

*(E)-3-(2,4-dimethylphenyl)-1-(4-((3-methylbut-2-en-1-yl)oxy)phenyl)prop-2-en-1-one* (**C12**), yellow solid (75% yield); mp 102–105 °C. ^1^H-NMR (400 MHz, CDCl_3_): δ 1.58–1.59 (m, 6H, 2 × CH_3_), 2.37 (s, 3H, CH_3_), 2.47 (s, 3H, CH_3_), 4.62 (d, *J* = 6.8 Hz, 2H, CH_2_) 5.51 (m, 1H, =CH), 7.00–7.09 (m, 4H, Ar), 7.47 (d, *J*_xy_ = 15.6 Hz, 1H, H_x_), 7.63 (d, *J* = 8.4 Hz, 1H, Ar), 8.06 (d, *J* = 9.2 Hz, 2H, Ar), 8.11 (d, *J*_xy_ = 15.6 Hz, 1H, H_y_).

*(E)-3-(2-chlorophenyl)-1-(4-((3-methylbut-2-en-1-yl)oxy)phenyl)prop-2-en-1-one* (**C13**), white solid (94% yield); mp 98–100 °C. ^1^H-NMR (400 MHz, CDCl_3_): δ 1.79 (s, 3H, CH_3_), 1.84 (s, 3H, CH_3_), 4.62 (d, *J* = 6.7 Hz, 2H, CH_2_), 5.52 (br, 1H, =CH), 6.99–7.03 (m, 2H, Ar), 7.33–7.36 (m, 2H, Ar), 7.45–7.48 (m, 1H, Ar), 7.53 (d, *J*_xy_ = 15.7 Hz, 1H, H_x_), 7.76–7.78 (m, 1H, Ar), 8.01 (d, *J* = 8.8 Hz, 1H, Ar), 8.05 (d, *J* = 8.8 Hz, 1H, Ar), 8.19 (d, *J*_xy_ = 15.6 Hz, 1H, H_y_).

*3-(3-chlorophenyl)-1-(4-((3-methylbut-2-en-1-yl)oxy)phenyl)prop-2-en-1-one* (**C14**), white solid (94% yield); mp 92–94 °C. δ 1.78 (s, 3H, CH_3_), 1.83 (s, 3H, CH_3_), 4.61 (m, 2H, CH_2_), 5.49 (br, 1H, =CH), 7.05–7.08 (m, 2H, Ar), 7.30–7.34 (m, 2H, Ar), 7.49–7.51 (m, 1H, Ar), 7.55 (d, *J*_xy_ = 15.7 Hz, 1H, H_x_), 7.74–7.72 (m, 1H, Ar), 8.00–8.02 (m, 1H, Ar), 8.03–8.05 (m, 1H, Ar), 8.21 (d, *J*_xy_ = 15.6 Hz, 1H, H_y_).

*(E)-3-(2,4-dichlorophenyl)-1-(4-((3-methylbut-2-en-1-yl)oxy)phenyl)prop-2-en-1-one* (**C16**), yellow solid (96% yield); mp 127–130 °C. ^1^H-NMR (400 MHz, DMSO-*d*_6_): δ 1.74 (s, 3H, CH_3_), 1.76 (s, 3H, CH_3_), 4.66 (d, *J* = 6.8 Hz, 2H, CH_2_), 5.44–5.47 (m, 1H, =CH), 7.09 (d, *J* = 9.2 Hz, 2H, Ar), 7.54–7.57 (m, 1H, Ar), 7.76 (s, 1H, Ar), 7.94 (d, *J*_xy_ = 15.2 Hz, 1H, H_x_), 8.04 (d, *J*_xy_ = 15.2 Hz, 1H, H_y_), 8.18 (d, *J* = 8.8 Hz, 2H, Ar), 8.27 (d, *J* = 8.8 Hz, 1H, Ar).

*3-([1,1′-biphenyl]-4-yl)-1-(4-((3-methylbut-2-en-1-yl)oxy)phenyl)prop-2-en-1-one* (**C17**), yellow solid (75% yield); mp 122–124 °C. ^1^H-NMR (400 MHz, DMSO-*d*_6_): δ 1.74 (s, 3H, CH_3_), 1.76 (s, 3H, CH_3_), 4.66 (d, *J* = 6.4 Hz, 2H, CH_2_), 5.46 (t, *J* = 6.8 Hz, 1H, =CH), 7.09 (d, *J* = 8.8 Hz, 2H, Ar), 7.39–7.52 (m, 3H, Ar), 7.75–7.79 (m, 5H, 4 × Ar + =CH), 7.98–8.03 (m, 3H, 2 × Ar + =CH), 8.19 (d, *J* = 8.8 Hz, 2H, Ar).

*(E)-1-(4-((3-methylbut-2-en-1-yl)oxy)phenyl)-3-(naphthalen-1-yl)prop-2-en-1-one* (**C18**), yellow solid (67% yield); mp 82–84 °C. ^1^H-NMR (400 MHz, CDCl_3_): δ 1.80 (s, 3H, CH_3_), 1.84 (s, 3H, CH_3_), 4.60–4.64 (m, 2H, CH_2_), 5.51–5.55 (m, 1H, =CH), 7.03 (d, *J* = 8.8 Hz, 2H, Ar), 7.54–7.61 (m, 3H, Ar), 7.66 (d, *J*_xy_ = 15.6 Hz, 1H, H_x_), 7.92–7.94 (m, 3H, Ar), 8.11 (d, *J* = 8.8 Hz, 2H, Ar), 8.28–8.30 (m, 1H, Ar), 8.68 (d, *J*_xy_ = 15.2 Hz, 1H, H_y_).

#### 3.2.2. Synthesis and Characterization Data for 3,5-disubstituted-*N*-thiocarbamoylated Pyrazolines **P1**–**P6**

Synthesis and characterization data for compounds **P1**–**P5** were in accordance with reference [26]. For the synthesis of compound **P6** we operated as follows: to a stirring solution of 1-(4-((3,7-dimethylocta-2,6-dien-1-yl)oxy)phenyl)ethan-1-one (1 eq.) and thiosemicarbazide (2.8 eq.) in ethanol (50 mL) was added dropwise a solution of ground potassium hydroxide (2.8 eq.) in hot ethanol (50 mL). The reaction was stirred at reflux for 48 h and then quenched with 200 mL of ice/water. The resulting suspension was extracted with chloroform (3 × 20 mL). The organics reunited were dried over sodium sulfate and evaporated in vacuo. Purification by column chromatography on silica gel (ethyl acetate–*n*-hexane 1:2) gave the title compound.

*3-(4-((3,7-dimethylocta-2,6-dien-1-yl)oxy)phenyl)-5-phenyl-4,5-dihydro-1H-pyrazole-1-carbothioamide* (**P6**), orange oil, 62% yield. ^1^H-NMR (400 MHz, CDCl_3_): δ 1.63 (s, 3H, CH_3_), 1.70 (s, 3H, CH_3_), 1.76 (s, 3H, CH_3_), 2.10–2.17 (m, 4H, 2 × CH_2_), 3.12 (dd, *J*_ab_ = 17.6 Hz, *J*_ac_ = 3.6 Hz, 1H, H_a_), 3.75 (dd, *J*_ab_ = 17.6 Hz, *J*_bc_ = 11.2 Hz, 1H, H_b_), 4.59 (d, *J* = 6.8 Hz, 2H, CH_2_), 5.10–5.13 (m, 1H, =CH), 5.48–5.51 (m, 1H, =CH), 6.02 (dd, *J*_bc_ = 11.2 Hz, *J*_ac_ = 3.6 Hz, 1H, H_c_), 6.52 (br, 1H, NH_2_, D_2_O exchange), 6.95 (d, *J* = 8.8 Hz, 2H, Ar), 7.15 (br, 1H, NH_2_, D_2_O exchange), 7.22–7.24 (m, 3H, Ar), 7.29–7.33 (m, 2H, Ar), 7.66 (d, *J* = 8.8 Hz, 2H, Ar). ^13^C-NMR (101 MHz, CDCl_3_): δ 16.7 (CH_3_), 17.8 (CH_3_), 25.7 (CH_3_), 26.3 (CH_2_), 39.6 (C(4)H_2_, pyr), 51.5 (CH_2_), 64.9 (CH_2_-O), 95.8 (C(5)H, pyr), 114.7 (2 × Ar), 119.4 (=CH), 123.9 (=CH), 125.3 (2 × Ar), 126.9 (2 × Ar), 128.9 (2 × Ar), 130.4 (Ar), 131.1 (C=), 131.8 (Ar), 136.3 (C=), 141.3 (Ar), 153.1 (C=N, pyr), 158.7 (Ar), 175.8 (C=S). Calcd. for C_26_H_31_N_3_OS: C, 72.02; H, 7.21; N, 9.69. Found: C, 72.18; H, 7.15; N, 9.61.

#### 3.2.3. Synthesis and Characterization Data for 3,5-disubstituted-N-acetylated Pyrazolines **P7**–**P18**

To a stirring solution of the proper chalcone (1 eq.) in acetic acid (15 mL) was added dropwise hydrazine hydrate (4 eq.). The reaction was stirred at reflux for 24–48 h and quenched with 200 mL of ice/water. The resulting suspension was extracted with chloroform (3 × 20 mL). The organics reunited were dried over sodium sulfate and evaporated in vacuo. Purification by column chromatography on silica gel with appropriate mixtures of ethyl acetate and *n*-hexane as mobile phase, gave the title compounds. Characterization data for compound **P8** were in accordance with [26].

*1-(3-(4-(allyloxy)phenyl)-5-phenyl-4,5-dihydro-1H-pyrazol-1-yl)ethan-1-one* (**P7**), orange solid (71% yield) mp 95–98 °C. ^1^H-NMR (400 MHz, DMSO-*d*_6_): δ 2.44 (s, 3H, CH_3_), 3.13 (d, *J*_ab_ = 17.4, Hz, *J*_ac_ = 4.6 Hz, 1 1H, H_a_), 3.71 (dd, *J*_ab_ = 17.6 Hz, *J*_bc_ = 12.0 Hz, 1H, H_b_), 4.36 (d, *J* = 5.2 Hz, 2H, CH_2_), 5.33 (d, *J*_cis_ = 11.2 Hz, 1H, =CH-H), 5.45 (d, *J*_trans_ = 17.2 Hz, 1H, =CH-H), 5.58 (dd, *J*_bc_ = 11.6 Hz, *J*_ac_ = 4.4 Hz, 1H, H_c_), 6.02–6.12 (m, 1H, =CH), 6.97 (d, *J* = 8.8 Hz, 2H, Ar), 7.24–7.26 (m, 3H, Ar), 7.32 (d, *J* = 7.2 Hz, Ar), 7.70 (d, *J* = 8.8 Hz, Ar). ^13^C-NMR (101 MHz, CDCl_3_): δ 22.0 (CH_3_), 42.5 (C(4)H_2_, pyr), 59.8 (C(5)H_2_, pyr), 68.9 (CH_2_-O), 114.9 (2 × Ar), 118.0 (=CH), 124.2 (Ar), 125.6 (2 × Ar), 127.6 (Ar), 128.2 (2 × Ar), 128.9 (2 × Ar), 132.78 (Ar), 142.0 (=CH_2_), 153.7 (C=N, pyr), 160.4 (Ar), 168.6 (C=O). Calcd. for C_20_H_20_N_2_O_2_: C, 74.98; H, 6.29; N, 8.74. Found: C, 74.77; H, 6.19; N, 8.65.

*1-(3-(4-((3-methylbut-2-en-1-yl)oxy)phenyl)-5-(o-tolyl)-4,5-dihydro-1H-pyrazol-1-yl)ethan-1-one* (**P9**), yellow solid (61% yield); mp 110–112 °C. ^1^H-NMR (400 MHz, CDCl_3_): δ 1.78 (s, 3H, CH_3_), 1.83 (s, 3H, CH_3_), 2.47 (s, 3H, CH_3_), 2.48 (s, 3H, CH_3_), 3.00 (dd, *J*_ab_ = 17.4 Hz, *J*_ac_ = 4.6 Hz, 1H, H_a_), 3.74 (dd, *J*_ab_ = 17.4 Hz, *J*_bc_ = 11.8 Hz, 1H, H_b_), 4.57 (d, *J* = 6.8 Hz, 2H, CH_2_), 5.52 (t, *J* = 6.8 Hz, 1H, =CH), 5.74 (dd, *J*_bc_ = 11.8 Hz, *J*_ac_ = 4.6 Hz, 1H, H_c_), 6.96 (d, *J =* 9.2 Hz, 2H, Ar), 7.01–7.03 (m, 1H, Ar), 7.13–7.20 (m, 3H, Ar), 7.69 (d, *J* = 8.8 Hz, 2H, Ar). ^13^C-NMR (101 MHz, CDCl_3_): δ 18.3 (CH_3_), 19.5 (CH_3_), 21.9 (CH_3_), 25.9 (CH_3_), 41.7 (C(4)H_2_, pyr), 57.0 (C(5)H_2_, pyr), 65.0 (CH_2_-O), 114.9 (2 × Ar), 119.2 (=CH), 123.9 (Ar), 124.0 (Ar), 126.6 (Ar), 127.4 (Ar), 128.2 (2 × Ar), 130.7 (Ar), 134.1 (Ar), 138.6 (C=), 139.9 (Ar), 153.8 (C=N, pyr), 160.7 (Ar), 168.5 (C=O). Calcd. for C_23_H_26_N_2_O_2_: C, 76.21; H, 7.23; N, 7.73. Found: C, 76.33; H, 7.37; N, 7.66.

*1-(3-(4-((3-methylbut-2-en-1-yl)oxy)phenyl)-5-(m-tolyl)-4,5-dihydro-1H-pyrazol-1-yl)ethan-1-one* (**P10**), orange solid (67% yield); mp 120–122 °C. ^1^H-NMR (400 MHz, CDCl_3_): δ 1.79 (s, 3H, CH_3_), 1.84 (s, 3H, CH_3_), 2.34 (s, 3H, CH_3_), 2.45 (s, 3H, CH_3_), 3.13 (dd, *J*_ab_ = 17.6 Hz, *J*_ac_ = 4.4 Hz, 1H, H_a_), 3.71 (dd, *J*_ab_ = 17.4 Hz, *J*_bc_ = 11.8 Hz, 1H, H_b_), 4.58 (d, *J* = 6.8 Hz, 2H, CH_2_), 5.51-5.54 (m, 1H, =CH), 5.55 (dd, *J*_bc_ = 11.6 Hz, *J*_ac_ = 4.4 Hz, 1H, H_c_), 6.97 (d, *J* = 8.4 Hz, 2H, Ar), 7.04–7.08 (m, 3H, Ar), 7.14–7.24 (m, 1H, Ar), 7.70 (d, *J* = 8.8 Hz, 2H, Ar). ^13^C-NMR (101 MHz, CDCl_3_): δ 18.3 (CH_3_), 21.5 (CH_3_), 22.0 (CH_3_), 25.9 (CH_3_), 42.5 (C(4)H_2_, pyr), 59.80 (C(5)H_2_, pyr), 65.0 (CH_2_-O), 114.9 (2 × Ar), 119.3 (=CH), 122.6 (Ar), 124.0 (Ar), 126.2 (Ar), 128.2 (2 × Ar), 128.4 (Ar), 128.8 (Ar), 138.5 (Ar), 138.6 (C=), 142.0 (Ar), 153.8 (C=N, pyr), 160.7 (Ar), 168.6 (C=O). Calcd. for C_23_H_26_N_2_O_2_: C, 76.21; H, 7.23; N, 7.73. Found: C, 76.33; H, 7.17; N, 7.85.

*1-(3-(4-((3-methylbut-2-en-1-yl)oxy)phenyl)-5-(p-tolyl)-4,5-dihydro-1H-pyrazol-1-yl)ethan-1-one* (**P11**), yellow oil (75% yield). ^1^H-NMR (400 MHz, CDCl_3_): δ 1.78 (s, 3H, CH_3_), 1.83 (s, 3H, CH_3_), 2.32 (s, 3H, CH_3_), 2.43 (s, 3H, CH_3_), 3.11–3.15 (m, 1H, H_a_), 3.67–3.74 (m, 1H, H_b_), 4.57 (d, *J* = 5.6 Hz, 2H, CH_2_), 5.52–5.58 (m, 1H, =CH + 1H, H_c_), 6.96 (d, *J* = 8.0 Hz, 2H, Ar), 7.10–7.14 (m, 4H, Ar), 7.68–7.70 (m, 2H, Ar). ^13^C-NMR (101 MHz, CDCl_3_): δ 18.3 (CH_3_), 21.1 (CH_3_), 22.0 (CH_3_), 25.9 (CH_3_), 42.5 (C(4)H_2_, pyr), 59.6 (C(5)H_2_, pyr), 64.9 (CH_2_-O), 114.9 (2 × Ar), 119.2 (=CH), 123.9 (Ar), 125.6 (2 × Ar), 128.2 (2 × Ar), 129.5 (2 × Ar), 137.2 (Ar), 138.7 (C=), 139.1 (Ar), 153.8 (C=N, pyr), 160.6 (Ar), 168.6 (C=O). Calcd. for C_23_H_26_N_2_O_2_: C, 76.21; H, 7.23; N, 7.73. Found: C, 76.01; H, 7.25; N, 7.88.

*1-(5-(2,4-dimethylphenyl)-3-(4-((3-methylbut-2-en-1-yl)oxy)phenyl)-4,5-dihydro-1H-pyrazol-1-yl)ethan-1-one* (**P12**), orange solid (97% yield); mp 117–119 °C. ^1^H-NMR (400 MHz, CDCl_3_): δ 1.78 (s, 3H, CH_3_), 1.83 (s, 3H, CH_3_), 2.28 (s, 3H, CH_3_), 2.43 (s, 3H, CH_3_), 2.47 (s, 3H, CH_3_), 3.00 (dd, *J*_ab_ = 17.4 Hz, *J*_ac_ = 4.6 Hz, 1H, H_a_), 3.74 (dd, *J*_ab_ = 17.4 Hz, *J*_bc_ = 11.8 Hz, 1H, H_b_), 4.57 (d, *J* = 6.4 Hz, 2H, CH_2_), 5.51 (t, *J* = 6.8 Hz, 1H, =CH), 5.71 (dd, *J*_bc_ = 11.6 Hz, *J*_ac_ = 4.4 Hz, 1H, H_c_), 6.89–7.01 (m, 5H, Ar), 7.68 (d, *J* = 8.8 Hz, 2H, Ar). ^13^C-NMR (101 MHz, CDCl_3_): δ 18.3 (CH_3_), 19.4 (CH_3_), 20.9 (CH_3_), 21.9 (CH_3_), 25.8 (CH_3_), 41.9 (C(4)H_2_, pyr), 56.8 (C(5)H_2_, pyr), 64.9 (CH_2_-O), 114.8 (2 × Ar), 119.2 (=CH), 124.0 (Ar), 124.1 (Ar), 127.3 (Ar), 128.2 (2 × Ar), 131.5 (Ar), 133.9 (Ar), 136.9 (Ar), 137.1 (Ar), 138.7 (C=), 153.8 (C=N, pyr), 160.6 (Ar), 168.6 (C=O). Calcd. for C_24_H_28_N_2_O_2_: C, 76.56; H, 7.50; N, 7.44. Found: C, 76.41; H, 7.41; N, 7.53.

*1-(5-(2-chlorophenyl)-3-(4-((3-methylbut-2-en-1-yl)oxy)phenyl)-4,5-dihydro-1H-pyrazol-1-yl)ethan-1-one* (**P13**), orange solid, (73% yield); mp 131–134 °C. ^1^H-NMR (400 MHz, CDCl_3_): δ 1.78 (s, 3H, CH_3_), 1.82 (s, 3H, CH_3_), 2.50 (s, 3H, CH_3_), 3.05 (dd, *J*_ab_ = 17.6 Hz, *J*_ac_ = 4.7 Hz, 1H, H_a_), 3.84 (dd, *J*_ab_ = 17.6 Hz, *J*_bc_ = 11.8 Hz, 1H, H_b_), 4.57 (d, *J* = 6.7 Hz, 2H, CH_2_), 5.50 (t, *J* = 6.7 Hz, 1H, =CH), 5.92 (dd, *J*_bc_ = 11.7 Hz, *J*_ac_ = 4.7 Hz, 1H, H_c_), 6.95 (d, *J* = 8.8 Hz, 2H, Ar), 7.07–7.09 (m, 1H, Ar), 7.21–7.23 (m, 2H, Ar), 7.40–7.42 (m, 1H, Ar), 7.68 (d, *J* = 8.8 Hz, 2H, Ar). ^13^C-NMR (101 MHz, CDCl_3_): δ 18.3 (CH_3_), 21.9 (CH_3_), 25.8 (CH_3_), 41.5 (C(4)H_2_, pyr), 57.6 (C(5)H_2_, pyr), 65.0 (CH_2_-O), 114.9 (2 × Ar), 119.3 (=CH), 123.7 (Ar), 125.9 (Ar), 127.3 (Ar), 128.2 (2 × Ar), 130.0 (Ar), 131.7 (Ar), 138.6 (Ar), 138.7 (C=), 154.1 (C=N, pyr), 160.8 (Ar), 168.7 (C=O). Calcd. for C_22_H_23_ClN_2_O_2_: C, 69.01; H, 6.06; N, 7.32. Found: C, 69.19; H, 6.13; N, 7.17.

*1-(5-(3-chlorophenyl)-3-(4-((3-methylbut-2-en-1-yl)oxy)phenyl)-4,5-dihydro-1H-pyrazol-1-yl)ethan-1-one* (**P14**), white solid, (61% yield); mp 85–88 °C. ^1^H-NMR (400 MHz, CDCl_3_): δ 1.78 (s, 3H, CH_3_), 1.83 (s, 3H, CH_3_), 2.45 (s, 3H, CH_3_), 3.12 (dd, *J*_ab_ = 17.6 Hz, *J*_ac_ = 4.4 Hz, 1H, H_a_), 3.74 (dd, *J*_ab_ = 17.6 Hz, *J*_bc_ = 12.0 Hz, 1H, H_b_), 4.58 (d, *J* = 6.4 Hz, 2H, CH_2_), 5.50 (d, *J* = 6.4 Hz, 1H, =CH), 5.55 (dd, *J*_bc_ = 12.0 Hz, *J*_ac_ = 4.4 Hz, 1H, H_c_), 6.97 (d, *J* = 8.8 Hz, 2H, Ar), 7.14 (d, *J* = 6.8 Hz, 1H, Ar), 7.22–7.29 (m, 3H, Ar), 7.69 (d, *J* = 8.8 Hz, 2H, Ar). ^13^C-NMR (101 MHz, CDCl_3_): δ 18.3 (CH_3_), 21.9 (CH_3_), 25.8 (CH_3_), 42.4 (C(4)H_2_, pyr), 59.4 (C(5)H_2_, pyr), 65.0 (CH_2_-O), 114.9 (2 × Ar), 119.2 (=CH), 123.6 (Ar), 123.9 (Ar), 125.8 (Ar), 127.9 (Ar), 128.2 (2 × Ar), 130.2 (Ar), 134.8 (Ar), 138.8 (C=), 143.9 (Ar), 153.8 (C=N, pyr), 160.8 (Ar), 168.8 (C=O). Calcd. for C_22_H_23_ClN_2_O_2_: C, 69.01; H, 6.06; N, 7.32. Found: C, 68.84; H, 5.99; N, 7.35.

*1-(5-(4-chlorophenyl)-3-(4-((3-methylbut-2-en-1-yl)oxy)phenyl)-4,5-dihydro-1H-pyrazol-1-yl)ethan-1-one* (**P15**), yellow solid (72% yield); 57–60 °C. ^1^H-NMR (400 MHz, CDCl_3_): δ 1.78 (s, 3H, CH_3_) 1.82 (s, 3H, CH_3_), 2.42 (s, 3H, CH_3_), 3.10 (dd, *J*_ab_ = 17.6 Hz, *J*_ac_ = 4.4 Hz, 1H, H_a_), 3.72 (dd, *J*_ab_ = 17.6 Hz, *J*_bc_ = 11.6 Hz, 1H, H_b_), 4.57 (d, *J* = 6.4 Hz, 2H, CH_2_), 5.49-5.51 (m, 1H, =CH), 5.54 (dd, *J*_ac_ = 4.4 Hz, *J*_bc_ = 11.6 Hz, 1H, H_c_), 6.96 (d, *J* = 8.8 Hz, 2H, Ar), 7.18 (d, *J* = 8.4 Hz, 2H, Ar), 7.29 (d, *J* = 8.8 Hz, 2H, Ar), 7.68 (d, *J =* 8.8 Hz, 2H, Ar). ^13^C-NMR (101 MHz, CDCl_3_): δ 18.3 (CH_3_), 21.9 (CH_3_), 25.8 (CH_3_), 42.3 (C(4)H_2_, pyr), 59.2 (C(5)H_2_, pyr), 65.0 (CH_2_-O), 114.9 (2 × Ar), 119.2 (=CH), 123.7 (Ar), 127.1 (2 × Ar), 128.2 (2 × Ar), 129.0 (2 × Ar), 133.3 (Ar), 138.7 (=C=), 140.5 (Ar), 153.7 (C=N, pyr), 160.8 (Ar), 168.7 (C=O). Calcd. for C_22_H_23_ClN_2_O_2_: C, 69.01; H, 6.06; N, 7.32. Found: C, 68.91; H, 6.05; N, 7.41.

*1-(5-(2,4-dichlorophenyl)-3-(4-((3-methylbut-2-en-1-yl)oxy)phenyl)-4,5-dihydro-1H-pyrazol-1-yl)ethan-1-one* (**P16**), white solid (80% yield); mp 128–130 °C. ^1^H-NMR (400 MHz, CDCl_3_) 1.77 (s, 3H, CH_3_) 1.82 (s, 3H, CH_3_), 2.49 (s, 3H, CH_3_), 2.99–3.04 (m, 1H, H_a_), 3.79–3.86 (m, 1H, H_b_), 4.56 (d, *J* = 6 Hz, 2H, CH_2_), 5.50 (br, 1H, =CH), 5.84–5.86 (m, 1H, H_c_), 6.94 (d, *J* = 8.4 Hz, 2H, Ar), 7.01 (d, *J* = 7.6 Hz, 1H, Ar), 7.20 (d, *J* = 7.2 Hz, 1H, Ar), 7.43 (s, 1H, Ar), 7.67 (d, *J* = 8.4 Hz, 2H, Ar). ^13^C-NMR (101 MHz, CDCl_3_): δ 18.3 (CH_3_), 21.9 (CH_3_), 25.9 (CH_3_), 41.4 (C(4)H_2_, pyr), 57.3 (C(5)H_2_, pyr), 64.9 (CH_2_-O), 114.9 (2 × Ar), 119.1 (=CH), 123.5 (Ar), 127.0 (Ar), 127.6 (Ar), 128.2 (2 × Ar), 129.8 (Ar), 132.4 (Ar), 133.8 (Ar), 137.3 (Ar), 138.8 (C=), 154.1 (C=N, pyr), 160.8 (Ar), 168.8 (C=O). Calcd. for C_22_H_22_Cl_2_N_2_O_2_: C, 63.32; H, 5.31; N, 6.71. Found: C, 63.17; H, 5.35; N, 6.76.

*1-(5-([1,1′-biphenyl]-4-yl)-5-methyl-3-(4-((3-methylbut-2-en-1-yl)oxy)phenyl)-4,5-dihydro-1H-pyrazol-1-yl)ethan-1-one* (**P17**), white solid (63% yield); mp 132–135 °C. ^1^H-NMR (400 MHz, CDCl_3_): δ 1.79 (s, 3H, CH_3_) 1.84 (s, 3H, CH_3_), 2.47 (s, 3H, CH_3_), 3.20 (dd, *J*_ab_ = 17.6 Hz, *J*_ac_ = 4.8 Hz, 1H, H_a_), 3.77 (dd, *J*_ab_ = 17.6 Hz, *J*_bc_ = 11.6 Hz, 1H, H_b_), 4.59 (d, *J* = 6.8 Hz, 2H, CH_2_), 5.51–5.54 (m, 1H, =CH), 5.65 (dd, *J*_ac_ = 4.6 Hz, *J*_bc_ = 11.8 Hz, 1H, H_c_), 6.98 (d, *J* = 8.8 Hz, 2H, Ar), 7.32–7.35 (m, 3H, Ar), 7.42–7.44 (m, 2H, Ar), 7.55–7.57 (m, 4H, Ar), 7.72 (d, *J* = 8.8 Hz, 2H, Ar). ^13^C-NMR (101 MHz, CDCl_3_): δ 18.3 (CH_3_), 22.0 (CH_3_), 25.9 (CH_3_), 42.4 (C(4)H_2_, pyr), 59.6 (C(5)H_2_, pyr), 65.0 (CH_2_-O), 114.9 (2 × Ar), 119.2 (=CH), 123.9 (Ar), 126.1 (2 × Ar), 127.1 (2 × Ar), 127.3 (Ar), 127.7 (2 × Ar), 128.2 (2 × Ar), 128.8 (2 × Ar), 138.7 (C=), 140.6 (Ar), 140.8 (Ar), 141.0 (Ar), 153.8 (C=N, pyr), 160.7 (Ar), 168.7 (C=O). Calcd. for C_28_H_28_N_2_O_2_: C, 79.22; H, 6.65; N, 6.60. Found: C, 79.41; H, 6.69; N, 6.45.

*1-(3-(4-((3-methylbut-2-en-1-yl)oxy)phenyl)-5-(naphthalen-1-yl)-4,5-dihydro-1H-pyrazol-1-yl)ethan-1-one* (**P18**), orange solid (77% yield); mp 78–80 °C. ^1^H-NMR (400 MHz, CDCl_3_): δ 1.75 (s, 3H, CH_3_), 1.84 (s, 3H, CH_3_), 2.45 (s, 3H, CH_3_), 3.19 (dd, *J*_ab_ = 17.7 Hz, *J*_ac_ = 4.2 Hz, 1H, H_a_), 3.77 (dd, *J*_ab_ = 17.4 Hz, *J*_bc_ = 11.7 Hz, 1H, H_b_), 4.55 (d, *J* = 6.9 Hz, 2H, CH_2_), 5.47–5.52 (m, 1H, =CH), 5.63–5.67 (dd, *J*_bc_ = 11.7 Hz, *J*_ac_ = 4.8 Hz, 1H, H_c_), 6.95 (d, *J* = 9.0 Hz, 1H, Ar), 7.31–7.34 (m, 1H, Ar), 7.40–7.45 (m, 2H, Ar), 7.68–7.74 (m, 3H, Ar), 7.77–7.81 (m, 4H, Ar). ^13^C-NMR (101 MHz, CDCl_3_): δ 18.3 (CH_3_), 22.0 (CH_3_), 25.9 (CH_3_), 42.5 (C(4)H_2_, pyr), 60.0 (C(5)H_2_, pyr), 65.0 (CH_2_-O), 114.9 (2 × Ar), 119.1 (=CH), 123.5 (Ar), 123.9 (Ar), 124.5 (Ar), 125.9 (Ar), 126.2 (Ar), 127.6 (Ar), 128.0 (Ar), 128.2 (2 × Ar), 129.0 (Ar), 132.9 (Ar), 133.3 (Ar), 138.8 (C=), 139.2 (Ar), 153.9 (C=N, pyr), 160.7 (Ar), 168.8 (C=O). Calcd. for C_26_H_26_N_2_O_2_: C, 78.36; H, 6.58; N, 7.03. Found: C, 78.21; H, 6.71; N, 6.99.

### 3.3. hMAO-A and hMAO-B Inhibition Studies

Microsomes from insect cells expressing recombinant hMAO-A or hMAO-B (5 mg protein/mL) served as enzyme sources, and were pre-aliquoted and stored at −70 °C. All enzymatic reactions were carried out to a final volume of 200 µL in black 96-well microtiter plates (Corning). Potassium phosphate buffer (100 mM, pH 7.4, made isotonic with KCl) served as reaction solvent. The reactions contained kynuramine (50 µM), various concentrations of the test inhibitor (0.003–100 μM), horseradish peroxidase (1 unit/mL) and Amplex^®^ Red (200 µM). Stock solutions of the test inhibitors were prepared in DMSO and added to the reactions to yield a final concentration of 4% (*v*/*v*) DMSO. The reactions were initiated with the addition of hMAO-A (0.0075 mg protein/mL) or hMAO-B (0.015 mg protein/mL), and incubated at 37 °C for 20 min in a convection oven. At endpoint, the reactions were terminated with the addition of 10 μL pargyline (20 mM) and 10 μL (*R*)-deprenyl (5 mM) for hMAO-A and hMAO-B, respectively. The concentrations of resorufin in the reactions were determined by fluorescence spectrophotometry (λ_ex_ = 560 nm, λ_em_ = 590 nm) [28]. Resorufin was quantitated with a linear calibration curve, which was constructed from standard solutions containing hydrogen peroxide (0.05–1.6 µM). Each standard was prepared to a final volume of 200 μL in potassium phosphate buffer and also contained horseradish peroxidase (1 unit/mL), Amplex^®^ Red (200 µM), 4% DMSO as co-solvent, 10 μL pargyline (20 mM) for hMAO-A and 10 μL (*R*)-deprenyl (5 mM) for hMAO-B. The IC_50_ values were determined by constructing sigmoidal curves of MAO catalytic activity versus the logarithm of inhibitor concentration. For this purpose, the one site competition model incorporated into the Prism 5 software package (GraphPad, San Diego, CA, USA) was used. All experiments were carried out in triplicate and the IC_50_ values are expressed as mean ± standard deviation (SD) [31].

### 3.4. Enantioselective HPLC

Semipreparative HPLC separations of the enantiomers of **P1**–**P5** were carried out on the commercially available 250 × 10 mm I.D. Chiralpak AD (Chiral Technologies Europe, Illkirch, France) column using pure ethanol elution mode. The temperature was set at 25 °C. The flow-rate was 2.5 mL min^−1^. HPLC apparatus consisted of a Perkin-Elmer (Norwalk, CT, USA) 200 LC pump equipped with a Rheodyne (Cotati, CA, USA) injector, a 5 mL sample loop, an HPLC Perkin-Elmer oven and a Perkin-Elmer detector. The signal was acquired and processed by Clarity software (DataApex, Prague, Czech Republic). The amounts of racemic samples resolved for single chromatographic runs ranged from 1 to 10 mg.

### 3.5. Molecular Modeling Studies

The crystal structures of hMAO-A (PDB code 2Z5X) and hMAO-B (PDB code 4A79) were taken from the Protein Data Bank [32]. Molecular docking calculations were performed with AUTODOCK 4.2 [33] subjected to a robust docking procedure already applied in virtual screening and pose prediction studies [34,35]. Autodock Tools were employed to identify the torsion angles in the ligands, add the solvent model and assign the Kollman atomic charges to the proteins. Ligand charges were calculated with the Gasteiger method. A grid spacing of 0.375 Å and a distance-dependent function of the dielectric constant were used for the energetic map calculations. Each docked compound was subjected to 200 runs of the AUTODOCK search using the Lamarckian Genetic Algorithm performing 10,000,000 steps of energy evaluation. The number of individuals in the initial population was set to 500 and a maximum of 10,000,000 generations were simulated during each docking run. All other settings were left as their defaults and the best docked conformations were taken into account. The selected docking poses were then refined through energy minimization in explicit water environment [36]. The ligand-protein complexes were minimized employing Amber 16 software (University of California, San Francisco, CA, USA) [37] with ff14SB force field. The complexes were placed in a rectangular parallelepiped water box, using the TIP3P explicit solvent model for water, and were solvated with a 15 Å water cap. Sodium ions were added as counter ions to neutralize the system. Two minimization stages consisting of 5000 steps of steepest descent followed by conjugate gradient, until a convergence of 0.05 kcal/Å mol, were then performed. In the first one, the protein was kept rigid with a position restraint of 100 kcal/mol·Å^2^ to uniquely minimize the positions of the water molecules. In the second stage, the entire system was energy minimized by applying a harmonic potential of 10 kcal/mol·Å^2^ only to the protein α carbons.

## 4. Conclusions

This research paper aimed to reinforce the privileged relationship between the pyrazoline scaffold and the hMAO inhibitory activity. These compounds were obtained through a well-established synthetic pathway. The substitution pattern at N1, C3 and C5 positions can finely modulate the inhibitory activity and isoform selectivity of these heterocyclic compounds. Bulky aromatic groups at C5 were not tolerated. *p*-Prenyloxyaryl moiety at C3 oriented the selectivity toward the B isoform. Moreover, single enantiomers obtained by HPLC enantioseparation were tested. The outcomes highlighted the importance of the C5 stereochemistry, displaying a better hMAO-B recognition of the (*R*)-enantiomers within this scaffold. In agreement with the experimental results, docking studies suggested that (*R*)-enantiomers would be able to form better H-bond interactions with respect to (*S*)-enantiomers and highlighted the limited space availability in the binding site region close to FAD cofactor, which may hamper the accommodation of excessively bulky groups at C5. Moreover, the bigger lipophilic pocket present in hMAO-B compared to that observed in hMAO-A may better welcome longer hydrophobic chains at C3, thus contributing to the selectivity of the corresponding ligands toward the B isoform.

## Figures and Tables

**Table 1:** Inhibitory activities (IC_50_) and selectivity index (SI) values of compounds **P1**–**P18** towards hMAO-A and hMAO-B.

Compound	Structure	IC_50_ (µM ± SD) ^a^		SI ^b^
		hMAO-A	hMAO-B	
(*S*)-**P1**	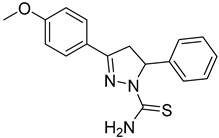	46.6 ± 2.62	>100	<0.47
(*R*)-**P1**	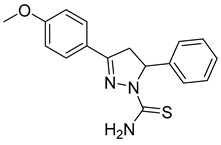	50.6 ± 2.67	>100	<0.51
(*S*)-**P2**	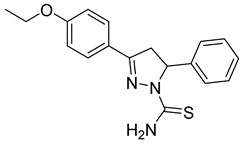	32.3 ± 4.46	>100	<0.32
(*R*)-**P2**	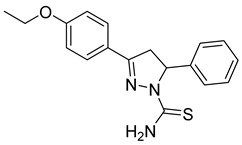	33.9 ± 1.29	>100	<0.34
(*S*)-**P3**	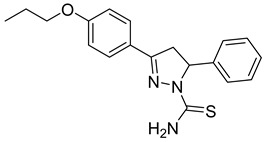	9.59 ± 0.83	42.5 ± 2.61	0.23
(*R*)-**P3**	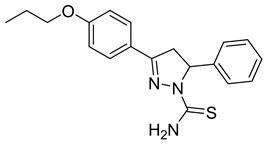	8.00 ± 0.83	2.77 ± 0.39	2.89
(*S*)-**P4**	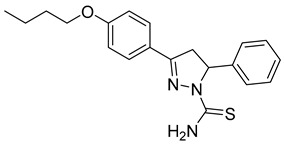	39.2 ± 2.15	5.03 ± 0.88	7.78
(*R*)-**P4**	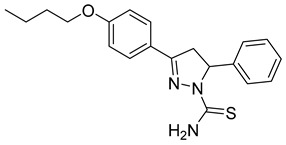	3.63 ± 0.50	0.38 ± 0.06	9.55
(*S*)-**P5**	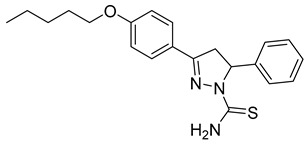	55.2 ± 2.72	2.79 ± 0.87	19.77
(*R*)-**P5**	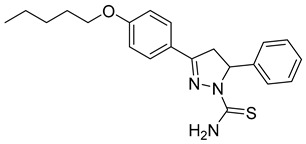	3.03 ± 0.60	0.44 ± 0.028	6.89
**P6**	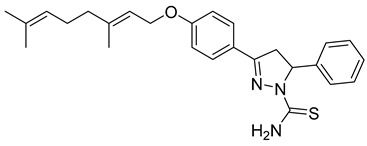	>100	54.1 ± 7.54	>1.85
**P7**	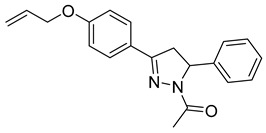	39.3 ± 5.94	14.5 ± 2.78	2.72
**P8**	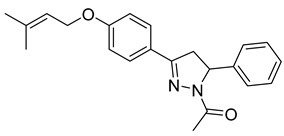	>100	2.29 ± 0.42	>43.67
**P9**	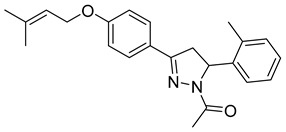	9.13 ± 0.34	27.3 ± 0.57	0.33
**P10**	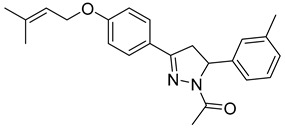	86.8 ± 14.7	3.22 ± 0.48	26.96
**P11**	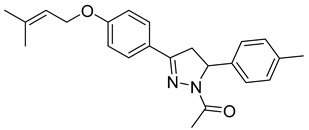	>100	12.2 ± 0.71	>8.19
**P12**	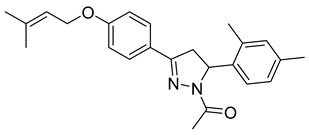	80.6 ± 10.0	>100	<0.81
**P13**	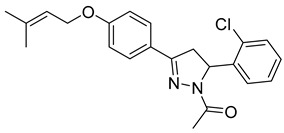	5.74 ± 1.66	3.11 ± 0.66	1.85
**P14**	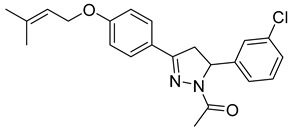	4.13 ± 0.26	1.08 ± 0.05	3.82
**P15**	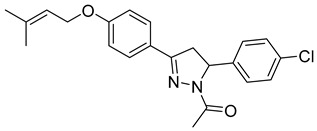	12.1 ± 0.14	11.0 ± 3.56	1.11
**P16**	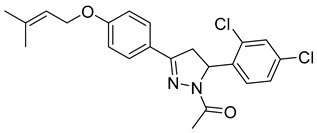	>100	>100	\
**P17**	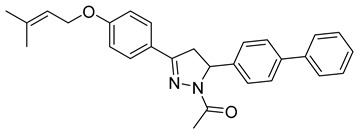	>100	>100	\
**P18**	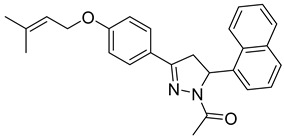	>100	>100	\
Toloxatone		3.92 ± 0.15 ^c^	-	-
Lazabemide		-	0.091 ± 0.15 ^c^	-

^a^ Values are the mean ± SD of triplicate determinations. ^b^ Selectivity index for the hMAO-B isoform, given as the ratio: IC_50_(hMAO-A)/IC_50_(hMAO-B). Values taken from [29].
